# Clot Formation in the Sipunculid Worm *Themiste petricola*: A Haemostatic and Immune Cellular Response

**DOI:** 10.1155/2012/280675

**Published:** 2012-03-25

**Authors:** Tomás Lombardo, Guillermo A. Blanco

**Affiliations:** Laboratorio de Inmunotoxicologia (LaITO), IDEHU, CONICET, Hospital de Clínicas “José de San Martín", Universidad de Buenos Aires (UBA), Avenida Córdoba 2351 Piso 2, Buenos Aires CP 1120, Argentina

## Abstract

Clot formation in the sipunculid *Themiste petricola*, a coelomate nonsegmented marine worm without a circulatory system, is a cellular response that creates a haemostatic mass upon activation with sea water. The mass with sealing properties is brought about by homotypic aggregation of granular leukocytes present in the coelomic fluid that undergo a rapid process of fusion and cell death forming a homogenous clot or mass. The clot structure appears to be stabilized by abundant F-actin that creates a fibrous scaffold retaining cell-derived components. Since preservation of fluid within the coelom is vital for the worm, clotting contributes to rapidly seal the body wall and entrap pathogens upon injury, creating a matrix where wound healing can take place in a second stage. During formation of the clot, microbes or small particles are entrapped. Phagocytosis of self and non-self particles shed from the clot occurs at the clot neighbourhood, demonstrating that clotting is the initial phase of a well-orchestrated dual haemostatic and immune cellular response.

## 1. Introduction

**Figure 1:**
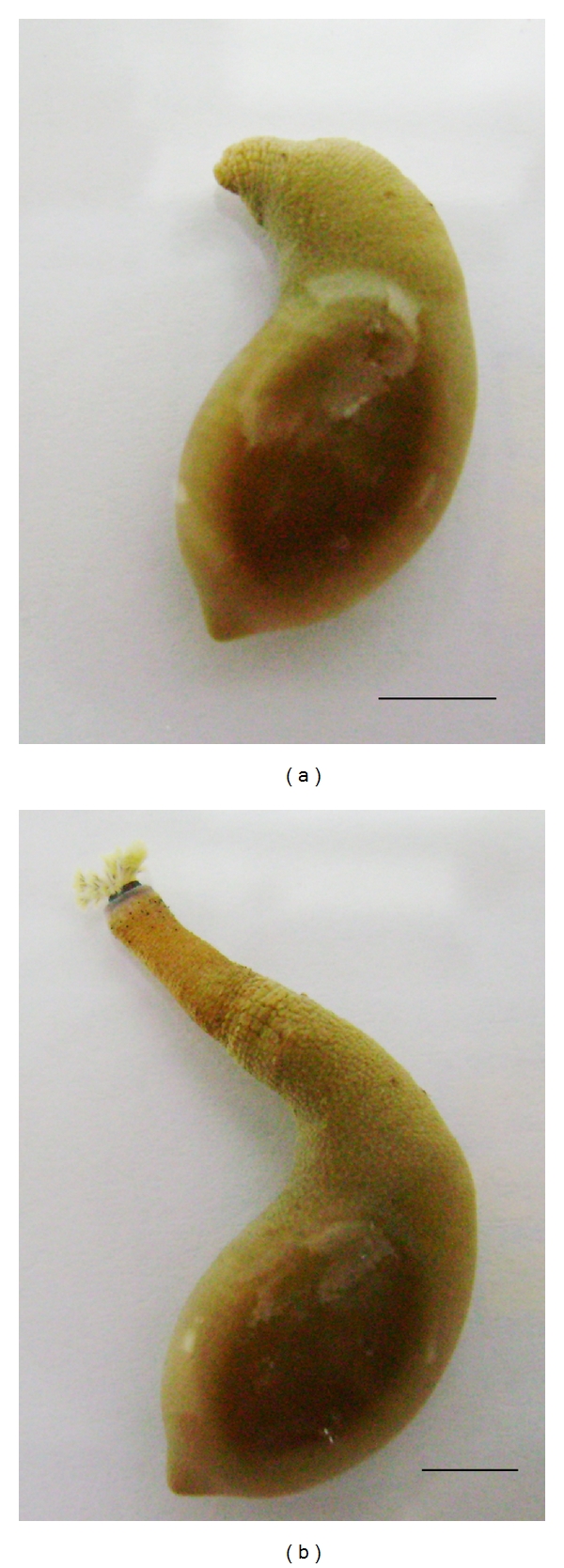
*Themiste petricola*, a species of the phylum Sipuncula, is shown in “peanut-shape” in (a) and in “worm-shape” in (b). Shape changes are due to contraction of the retractor and longitudinal muscles in (a), and relaxation of retractor and contraction of circular body wall muscles in (b). Intracoelomic pressure is higher in peanut-shape, and coelomic fluid may be strongly expelled if the body wall is ruptured while the worm is kept in this shape.

Sipunculans are a phylum of nonsegmented peanut-shaped marine worms that lack a true circulatory system [1]. Recent molecular phylogenetic analyses suggest a close relationship between Sipuncula and the phylum Annelida, particularly with the major group Polychaeta that includes mostly marine worms [2–4]. These worms have a coelomic cavity filled with cells in suspension enclosed by a muscular body wall (Figure 1). The coelomic cavity serves as a hydroskeleton and is lined by a peritoneum, surrounded by a muscular layer, a dermis, an epidermis, and a cuticle [5]. In some species the coelomic cavity has a series of canals that penetrate the body wall toward the dermis while in other species these canals form an interconnected network providing a comprehensive system for coelomic fluid circulation [1, 5].

Although several studies of coelomic cells have been conducted in species of Sipuncula for more than a hundred years, the presence of a clotting system has not been demonstrated until recently [6]. Comprehensive reviews on the phylum [1, 5] do not mention coelomic fluid clotting and despite some occasional references to clot masses made by a few authors [7, 8], it has been implicit that a clotting system was absent, perhaps due to the fact that jellification of cell-free coelomic fluid and formation of extracellular strands or fibres have never been observed.

Sipunculans have a slender retractile introvert ending in a mouth with tentacles that can be extruded or pulled back from the body trunk through a variable number of retractor muscles [1, 5, 9, 10]. When longitudinal body wall muscles and retractor muscles are contracted, the worm adopts a peanut shape, and when they are relaxed, the introvert is extruded and the worm shape is more apparent (Figure 1) [5]. Coelomic cavity pressures range from less than 1 cm of body fluid in the worm-shape to more than 50 cm in peanut shape, and even higher values during borrowing activities [11]. This variation in coelomic cavity pressure is the main factor causing coelomic fluid flow [5, 11]. If the body wall is damaged when the worm is in peanut shape, the coelomic fluid is expelled, the hydroskeleton function is lost, and the introvert retractor muscles can no longer work. In contrast, if the body wall is breached when the introvert is relaxed (e.g., an anesthetized animal), coelomic fluid loss is preserved. These facts underscore that coelomic fluid preservation is critical to the worm upon body wall injury, but its sessile aquatic lifestyle and the lack of a true circulatory system could have influenced the acquisition of a haemostatic mechanism not readily comparable to that of invertebrates with open circulatory system such as arthropods [12].

## 2. The Clotting System of *Themiste petricola*

**Figure 2:**
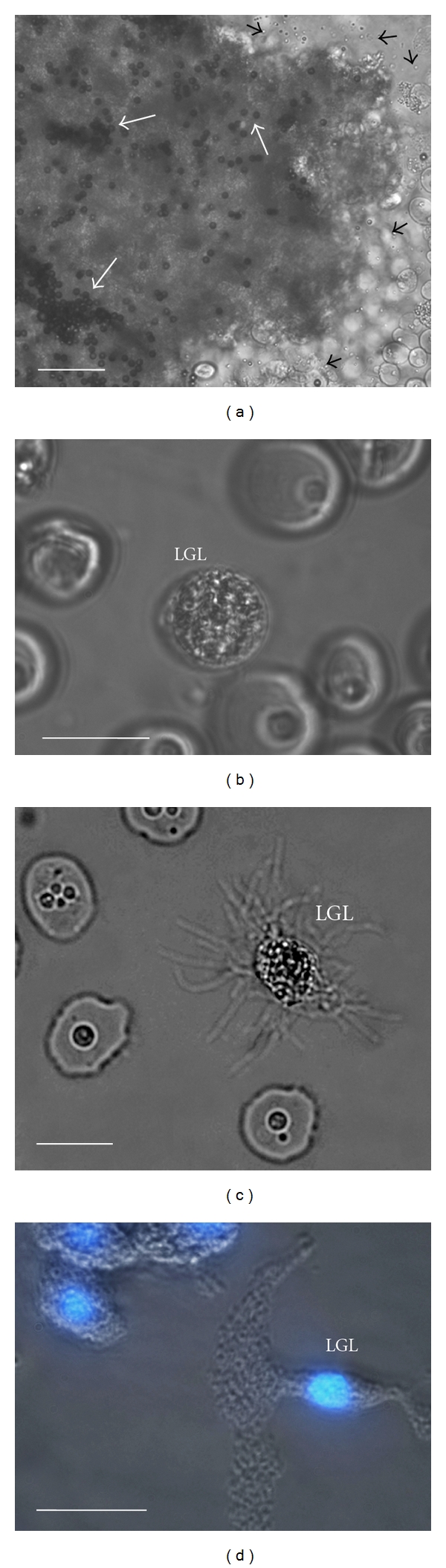
(a) A large clot formed by the aggregation of large granular leukocytes (LGLs) is shown entrapping magnetic beads (thick white arrows). These cellular clots are rapidly formed by contact with sea water and may serve a haemostatic purpose precluding loss of coelomic fluid upon body wall injury but may also serve an immune function entrapping foreign agents. The preparation corresponds to a male worm and numerous activated spermatozoids can be seen interspersed all around the microscopic field (thin black arrows). *Bar* 50 *μ*m. (b) A large granular leukocyte (LGL) is shown in resting state as observed when coelomic fluid is harvested using EDTA-containing saline solutions. *Bar* 15 *μ*m. (c) The presence of sea water or Ca++ containing saline induces massive morphological changes that include the extrusion of filopodia. *Bar* 15 *μ*m. (d) Activated LGLs adhere to each other to form a clot but may also adhere firmly and spread over glass surface acquiring very peculiar shapes. However, cell death and cytoplasmic disintegration of glass-adhered LGLs ensues within minutes, and samples must be fixed quickly in order to be observed microscopically. Phase contrast image digitally overlaid to a fluorescent image of a DAPI-stained preparation. *Bar* 15 *μ*m.

*Themiste petricola* (Amor, 1964) is a sipunculid worm that lives borrowed in rocks at intertidal areas [13, 14]. When coelomic fluid of an adult worm is harvested and exposed ex vivo to sea water, a group of specific cells become rapidly activated, aggregate homotypically, and create an insoluble mass that can be seen macroscopically [15, 16]. The haemostatic significance of this mass was demonstrated experimentally by its ability to block coelomic fluid flow [6]. When coelomic fluid was allowed to flow through a thin glass vessel connected in one open end to sea water, a macroscopic mass was formed at the site of contact with sea water, and the coelomic fluid column was retained upstream of the clot [6]. At the microscopic level, the clot mass is formed by a tight mass of aggregated cells (Figure 2(a)), contrasting with descriptions in arthropods where the clot is formed mainly by a network of extracellular strands with occasional cells interspersed [17, 18]. Clotting in *Themiste petricola* also accomplishes an immune role by entrapping microbes and other dissimilar particles within the clot mass (Figure 2(a)) [15, 16]. Massive clots can be obtained ex vivo by allowing coelomic fluid of a whole worm to clot over a suspension of magnetic beads containing small amounts of sea water that can be further separated with a magnet [6, 15, 16, 19]. Smaller clots can be formed by placing smaller aliquots of coelomic fluid mixed with small amounts of sea water over a glass surface. When coelomic fluid is placed over suspensions of bacteria or other foreign particles, these small clots are formed immediately, entrapping the particles and macroscopically resembling an agglutination reaction (Figure 4(a)) [6, 16].

**Figure 3:**
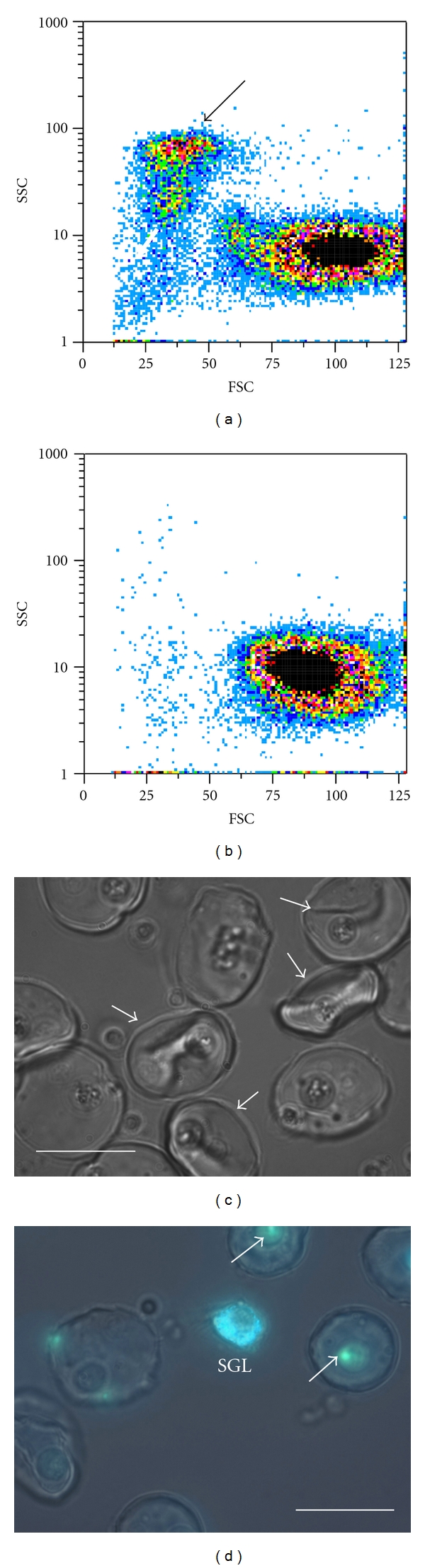
(a) Flow cytometry of coelomic cells. Forward light scatter (FSC) versus side light scatter (SSC) dot plot of a sample harvested in EDTA-containing saline. The cluster of LGLs is indicated by the arrow. The large cluster in lower-right position corresponds to haemerythrocytes and large hyaline amebocytes and accounts for more than 90% of all cells in the sample. (b) A similar dot plot corresponding to coelomic fluid from another worm harvested in Ca++ containing saline. The sample is depleted of LGLs due to activation, adhesion, and exclusion of the clotted cells by filtration through a 30 *μ*m mesh. (c) The non-clotting haemerythrocytes (arrows) are the most abundant cells in the coelomic fluid, have a characteristic biconcave disk shape, often have a single large acid vacuole, and are red coloured due to the presence of the respiratory pigment haemerythrin. *Bar* 15 *μ*m. (d) Large hyaline amebocytes (LHA) also have a single or a few large acid vacuoles and are actively phagocytic. In the photograph LHA can be observed with DAPI-stained bacteria ingested within a large acid vacuole (arrows). Small granular leukocytes (SGLs) are very active phagocytes. The cytoplasm of SGL in the figure is seen densely packed with phagocytosed DAPI-stained bacteria (phase contrast and fluorescent images were digitally overlaid). *Bar* 15 *μ*m.

**Figure 4:**
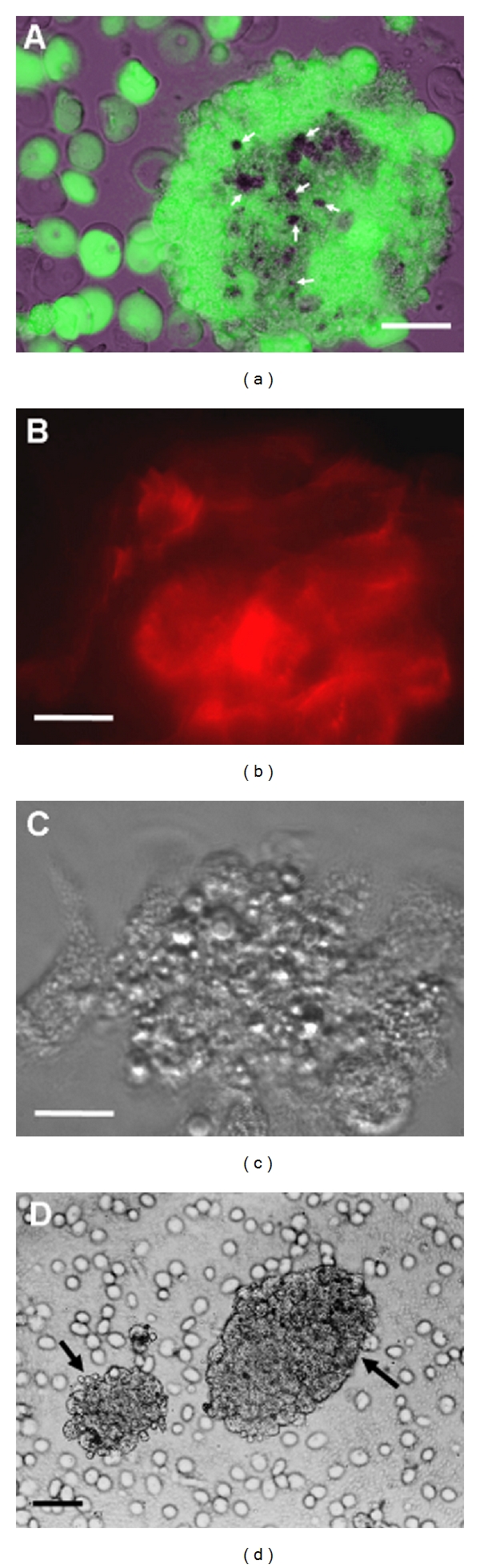
(a) A small clot-entrapping bacteria (arrows). Lysosome rupture, cell death, and nucleic acid degradation occur first in the inner parts of the clot creating a hostile degradative environment for the captured pathogens. Green fluorescence corresponds to viable cells as indicated by fluorescein-diacetate (FDA) probe. The dark area in the centre of the clot is due to the abundance of dead cells which do not retain FDA. Nonclotting phagocytic cells (LHAs and SGLs; shown in Figure 3(d)) are found in the neighbourhood and have an ancillary role engulfing self and foreign material detached from the clot. Phase contrast and fluorescent images were digitally overlaid. *Bar* 30 *μ*m. (b) The clot hardness is brought by preserving F-actin after death of adhered cells. The insoluble mesh of F-actin is detected by staining with red-fluorescent probe phalloidin rhodamine. *Bar* 15 *μ*m. (c) Phase contrast image of the clot shown in (b). *Bar* 15 *μ*m. (d) When particles are injected in vivo, several small clots (arrows) with a size comparable to that of female ovocytes or male clusters of maturing spermatic cells are formed instead of a massive single clot as occurs ex vivo. This may facilitate extrusion of entrapped material through the nephridia. *Bar* 50 *μ*m.

There is no standardized nomenclature of sipunculan coelomic cells, and many differences seem to occur between species. The aggregating cells that form the clot in *Themiste petricola* have been designated large granular leukocytes (LGLs) (Figure 2). These clotting cells are notably similar to descriptions of type I granulocytes in *Sipunculus nudus* [20]. Since coelomic fluid is mainly a single-cell suspension, it is quite suitable to flow cytometry analysis [21]. Harvesting coelomic cells in EDTA-containing solutions prevents adhesion of LGLs and allows analysis and quantification of these cells by flow cytometry (Figure 3(a)). Resting LGLs are found as a single cluster with high side light scatter due to its granular content [16]. By light and fluorescence microscopy resting LGLs appear as regularly round and coarsely granular cells (Figure 2(b)). Granules have acid content and can be stained with supravital lysosomotropic probes like acridine orange [16]. As demonstrated by flow cytometry analysis, if coelomic fluid is harvested and maintained in sea water or Ca++ containing solutions, the cluster of resting LGLs disappears and only nonclotting coelomic cells are retained (Figure 3(b)) [16]. Thus studies of sipunculan coelomocytes must consider that a harvesting medium made of sea water or Ca++ containing saline solutions will deplete coelomic fluid of LGLs. Quantification by flow cytometry showed that nonclotting cells represent the majority of cells in suspension being about 92% of the total count [16]. Thus LGLs are a relatively low fraction of coelomic cells. Among non-clotting cells haemerythrocytes, carrying the respiratory pigment haemerythrin, is the most abundant cell type as occurs in all sipunculan species (Figure 3(c)) [1, 5, 7]. Other non-clotting cells found in *Themiste petricola* and involved in immune reactions are small granular leukocytes (SGL; Figure 3(d)) and large hyaline amebocytes (LHA; Figure 3(d)). LHAs and SGLs have an important role in assisting the immune purpose of clot formation (Figure 4(a)) [6].

## 3. The Process of Clot Formation and the Clot Structure at the Cellular Level

Experimental small clots formed over glass surface by placing small aliquots of coelomic fluid with controlled amounts of sea water are useful in evaluating morphological changes of LGLs following activation. Extrusion of filopodia (Figure 2(c)), large pseudopodia, cell-cell adhesion and fusion, partial or total degranulation, and formation of the most curious cell shapes can be observed in activated LGLs (Figure 2(d)) [15, 16]. Small clots formed experimentally and consisting of several LGLs aggregated to form a multicellular spheroid make apparent the clot structure and the significance of LGL activation and aggregation (Figure 4(a)). The central areas of the clot show fusion of cells, massive release of acid granules content, and degradation of nuclei and DNA content [6, 16]. The peripheral areas of the clot often show LGLs still having acid granules and preserved nuclei [6, 16]. Supravital staining to assess viability demonstrates that LGL death occurs in the whole clot although it appears to occur first or more rapid at the inner zones (Figure 4(a)) [6, 15, 16]. Supravital assessment with fluorescent probes has shown some basic characteristics of clot components in *Themiste petricola*. Lipophilic dyes showed a huge amount of lipid content which is consistent with good sealing properties, sulforhodamine B demonstrated permeation of LGLs as occurs in activated mammalian platelets, and Annexin V demonstrated phosphatidylserine exposure [6, 16]. In fixed samples fluorescent-labelled phalloidin demonstrated that a mesh of F-actin derived from aggregated and fused LGLs creates a massive scaffold of fibrous protein where lipids and other LGL-derived content are retained (Figures 4(b) and 4(c)) [6]. Thus, unlike most commonly known mechanism of programmed cell death where F-actin is actively disassembled [22, 23], during LGL death and clot formation a syncytial F-actin cytoskeleton is assembled after cell-cell adhesion, and it is preserved upon LGL massive death. This large supracellular arrangement of insoluble fibrous actin may be crucial in determining the clot structure and conferring sealing properties (Figures 4(b) and 4(c)). In jelly-like clots occurring in arthropods and higher vertebrates extracellular strands of polymerized insoluble proteins form the main clot structure that is additionally strengthened by the crosslinking activity of transglutaminases [17, 18, 24–28]. Either platelet-derived or coagulocyte-derived components are retained within the mesh of extracellular strands [17, 18, 29]. By retaining lipids and other LGL-derived material, the insoluble scaffold of F-actin may achieve a similar mechanical sealing result in the peculiar clotting system of the sipunculid *Themiste petricola*.

Tissue transglutaminase is a Ca++-dependent enzyme that crosslinks cytoskeletal proteins during end stages of apoptosis and contributes to prevent leakage of potentially harmful cell remnants [30–32]. For example, shedding of cytoplasmic and nuclear remnants, under the form of cytoplasmic microvesicles or DNA containing microparticles, during cell death of placental multinucleated syncytiotrophoblast is associated with preeclampsia [33–35]. Transglutaminase was shown to normally crosslink F-actin during cell death of multinucleated syncytiotrophoblast creating a large scaffold of polymerized actin that retained cell remnants of dead syncytium masses and prevented shedding of microvesicles [36]. It would be of interest to evaluate if a similar cross-linking system based on transglutaminase is present in LGLs and if it contributes to harden the F-actin scaffold of the clot and retain LGL remnants within the clot structure. 

## 4. Immune Aspects of Clot Formation

Clotting in *Themiste petricola* entraps dissimilar non-self particles within the clot mass but not self ovocytes, spermatic cells or other coelomic cell types. Thus, clot formation in sipunculans involves non-self recognition and is a first line immune reaction [37]. Several additional findings are consistent with the immune role of clotting. These include release of the content of LGL acid granules and massive degradation of nucleic acids, more noticeable at inner areas of the clot, and the fact that proteoglycan recognition protein small (PGRP-S) is present in the clot mass [16]. PGRP-S is a conserved pattern recognition protein with a relevant role in invertebrate innate immunity [38]. PGRP-S is highly expressed in resting LGLs and is also found at high levels in the clot supernatants when the reaction is elicited ex vivo [16].

Clotting in *Themiste petricola* was demonstrated to be part of a broader cellular response that extends to the clot neighbourhood. Fluorescently stained heat-killed bacteria were entrapped within the clot and were further observed to be phagocytosed by SGLs and LHAs at the clot neighbourhood (Figure 3(d)) [6]. Particularly single SGLs were often found in close proximity to the clot margins. By creating small clots over glass, it was observed that LGL activation ended in cytoplasmic fragmentation and formation of numerous regularly round remnants having a microvesicle-like shape of less than 2 *μ*m [6, 16]. These microvesicles were phagocytosed by SGLs and LHAs and the same occurred with bacteria. Evidence of shedding of nuclei remnants was obtained by creating clots in the presence of nonpermeant fluorescent DNA dyes, which cannot stain DNA in live cells but stain nuclear remnants from dead cells provided that DNA is not completely degraded. Under these conditions DNA label was found in high amounts within the cytoplasm of phagocytes at the clot neighbourhood indicating active phagocytosis of nuclear remnants shed from the clot [6]. A similar image of phagocytes having the cytoplasm filled with fragmented DNA was obtained by the nick-end DNA fluorescent labelling technique (TUNEL) [6]. Thus, cellular responses of SGLs and LHAs play the role of a second line of host defence in close connection to the clot structure but located at the clot vicinity.

As mentioned above, a massive macroscopic clot can be isolated ex vivo by allowing all coelomic fluid from an adult worm to clot over magnetic beads and further separating the clot with a magnet (Figure 2(a)). However, if the magnet beads are injected directly into the coelomic cavity of a worm and the fluid is harvested after 24 h, a massive clot is not recovered but instead the product obtained is several smaller clots entrapping beads (Figure 4(d)) [15]. These smaller clots made of aggregated LGLs (Figure 4(d)) are similar to descriptions of multicellular structures (brown bodies) made by several authors in some species of sipunculans [5, 20]. In contrast to arthropods where nodules remain in the hemocoel [18], multicellular structures entrapping foreign material may be expelled through the nephridia out of the coelomic cavity [5, 8, 39].

## 5. Clotting in *Themiste petricola* and Wound Repair in *Sipunculus nudus*

A recent study in *Sipunculus nudus* evaluated the course of histological changes after inducing experimental wounds in the body wall under controlled conditions [40]. Results of this study demonstrated several coincidences with experimental findings in the clotting system of *Themiste petricola*. Type I granulocytes of *Sipunculus nudus* (which are similar to LGLs) were the cells found at earlier time points at the site of injury, surrounding or partially immersed in an acidophilic mass. This mass created a soft haemostatic plug that contributed to prevent gush of coelomic fluid through the wound [20, 40]. Cell-shape changes such as spreading and elongation were also observed in type I granulocytes. The acidophilic material continued to increase during the first 15 h and contributed to the initial sealing of the injured body wall where muscles, dermis, and epidermis layers were experimentally breached [40]. The study demonstrated that at 24 h the wound was completely closed by acidophilic material and type I granulocytes [40]. The author hypothesized that acidophilic material could have been derived from degranulation of type I granulocytes [40]. However, the similarity of the histological description with LGL clotting by aggregation and cell death in *Themiste petricola* [6] suggests that the acidophilic material acting as an insoluble plug should be the clot itself in *Sipunculus nudus,* made of the insoluble remains of fused and dead Type I granulocytes together with the content released from acid granules. It also highlights that the rapid and massive cell death and degradation of granulocytes transforming themselves into a mass is a novel concept in sipunculan immunology and haemostasis, and that it should be considered in future experimental approaches of sipunculan coelomic cells. The study further showed that at later time points in wound healing a second type of granulocyte designated type II granulocyte was found to increase progressively in number and was located underneath of type I granulocytes and acidophilic material [40]. Type II granulocytes were described as phagocytic and are coincident in size morphology and overall histological description to that of SGLs of *Themiste petricola* [6, 20, 40]. After 24 h connective tissue was detected at the wound closure site, and by 96 h more collagen fibrils were observed and a new cuticle was formed [40]. Thus, the study provides the first indication that clotting by LGLs (Type I granulocytes in *Sipunculus nudus*) may be also involved in the first phase of wound repair.

## 6. Conclusion

The clotting system of the sipunculan *Themiste petricola* is based on activation, aggregation, and a peculiar form of programmed cell death of LGLs occurring within minutes. Nonclotting cells in contrast remain viable and engulf cytoplasmic and nuclear remnants of dead LGLs at the clot neighbourhood. The clot has both haemostatic and immune functions because it entraps particles during assemblage of the clot mass and creates a degradative environment within its interior, while retaining antibacterial pattern recognition proteins like PGRP-S. At sites of body wall injury, the clotting system will serve haemostatic, immune and wound repair functions. Within the coelom the system will serve predominantly immune functions entrapping microbes, facilitating phagocytosis, and potentially enabling massive extrusion of small size clots through the nephridia.
